# No impact of IL28B polymorphisms on liver enzymes in patients coinfected with HIV and HCV

**DOI:** 10.1186/1758-2652-13-S4-P206

**Published:** 2010-11-08

**Authors:** JV Fernández-Montero, L Martín-Carbonero, P Barreiro, NI Rallón, JM Benito, J González-Lahoz, V Soriano

**Affiliations:** 1 Hospital Carlos III, Infectious Diseases, Madrid, Spain; 2 Department of Infectious Diseases. Hospital Carlos III, C/ Sinesio Delgado 10, Madrid, Spain

## Background

IL-28B single nucleotide polymorphisms (SNPs) strongly influence both spontaneous HCV clearance and response to peginterferon-ribavirin therapy. There is no information about the impact of IL28B SNPs on the natural history of HCV liver disease and/or the rate of elevated liver enzymes.

## Methods

A cohort of HIV/HCV coinfected individuals with normal (<41 IU/L) or elevated (41 IU/L) ALT levels for >12 months were screened for the rs12979860 SNP at the IL-28B gene. The proportion of patients with the favorable (CC) or unfavorable (CT/TT) genotypes were compared in both groups.

## Results

A total of 124 patients (44% normal ALT levels, median age 42 years, 68% males, 93% IDUs, 33% alcohol abuse, 5% HBsAg+, median CD4 count 511 cells/µL, median serum HCV-RNA 6.05 log10 copies/mL, 62% HCV genotype 1) were analyzed. Overall 34% of the whole population displayed the IL-28B CC genotype. When comparing ALT groups, 18 (32.7%) with normal ALT showed CC vs 25 (36.2%) with elevated ALT (p=0.71). Using elastometry (FibroScan), liver fibrosis estimates were significantly lower at baseline in patients with normal vs elevated ALT (6.3±2 vs 14.4±12 kPa, respectively, p<0.001). Other differences amongst groups were not significant, as follows: baseline serum HCV-RNA (5.95 vs 6.05 log10 IU/mL, p=0.62), CD4 counts (499 vs 543 cells/µl, p=0.34), and prothrombin activity (91% in both groups, p=0.99). Patients with normal vs elevated ALT were found to be coinfected more frequently with HCV genotypes 1 or 4 (45% vs 26%, p=0.02).

## Conclusions

IL28B genotypes do not influence ALT levels in HIV-HCV coinfected patients. Higher ALT levels are associated with a greater extent of liver fibrosis.

